# The Role of Serotonergic Gene Methylation in Regulating Anxiety-Related Personality Traits in Chimpanzees

**DOI:** 10.3390/biology11111673

**Published:** 2022-11-17

**Authors:** Nicky Staes, Elaine E. Guevara, William D. Hopkins, Steven J. Schapiro, Marcel Eens, Chet C. Sherwood, Brenda J. Bradley

**Affiliations:** 1 Center for the Advanced Study of Human Paleobiology, Department of Anthropology, The George Washington University, Washington, DC 20052, USA; 2 Behavioural Ecology and Ecophysiology Group, Department of Biology, University of Antwerp, 2610 Antwerp, Belgium; 3 Centre for Research and Conservation, Royal Zoological Society of Antwerp, Koningin Astridplein 26, 2018 Antwerp, Belgium; 4 Michale E. Keeling Center for Comparative Medicine and Research, The University of Texas MD Anderson Cancer Center, Bastrop, TX 78602, USA

**Keywords:** Illumina Infinium Methylation EPIC array, rearing, behavioral style, primate

## Abstract

**Simple Summary:**

Serotonin is a neurotransmitter known to regulate psychological health (depression, anxiety) and personality in humans. External factors such as early life stress or medication use can impact the serotonin system, for example, through epigenetic modification of the genes that code for its different components. While human studies are currently exploring the effects of serotonergic methylation, an example of epigenetic modification, primate studies are largely lacking. In this study, we investigated to what extent personality traits in captive chimpanzees are associated with methylation levels of two genes that play a major role in serotonin transmission: the gene coding for its receptor subtype 1A (*HTR1A*) and the gene coding for its transporter (*SLC6A4*). Using a methylation array identical to human studies, we show that methylation levels are associated with variation in four chimpanzee personality traits linked with a reduction in anxiety and aggression and increase in prosocial and explorative behavior. Different from human studies, early atypical social rearing conditions only had a minor impact on methylation. The results from this study highlight evolutionarily conserved methylation sites that can be targeted in future hypothesis-driven studies across species.

**Abstract:**

While low serotonergic activity is often associated with psychological disorders such as depression, anxiety, mood, and personality disorders, variations in serotonin also contribute to normal personality differences. In this study, we investigated the role of blood DNA methylation levels at individual CpG sites of two key serotonergic genes (serotonin receptor gene 1A, *HTR1A*; serotonin transporter gene, *SLC6A4*) in predicting the personalities of captive chimpanzees. We found associations between methylation at 9/48 CpG sites with four personality dimensions: Dominance, Reactivity/Dependability, Agreeableness, and Openness. Directionality of effects were CpG location-dependent and confirmed a role of serotonergic methylation in reducing anxiety (Dominance) and aggression-related personality (Reactivity/Undependability) while simultaneously promoting prosocial (Agreeableness) and exploratory personalities (Openness). Although early-life adversity has been shown to impact serotonergic methylation patterns in other species, here, atypical early social rearing experiences only had a modest impact on CpG methylation levels in this chimpanzee sample. The precise environmental factors impacting serotonergic methylation in chimpanzees remain to be identified. Nevertheless, our study suggests a role in shaping natural variation in animal personalities. The results of this study offer a basis for future hypothesis-driven testing in additional populations and species to better understand the impact of ecology and evolution on complex behavioral traits.

## 1. Introduction

Serotonin (5-hydroxythalamine or 5-HT) is a chemical produced by nerve cells that has an impact on almost every physiological process throughout the body, including the gastrointestinal, cardiovascular, and central nervous systems [1]. Reduced serotonin activity plays a major role in the development of a number of psychiatric disorders that are highly prevalent in modern human populations, including depression, anxiety, and mood and personality disorders [1,2]. The levels of 5-HT that are available for neural signaling and regulate its activity are dependent on several factors such as the availability of the rate-limiting enzyme tryptophan hydroxylase-2 (TPH2), the number of serotonin reuptake transporters (5-HTT), and the number of available serotonin receptors [1]. For example, patients with depression typically show increased expression levels of serotonin receptor subtype 1A (HTR1A), and treatment of depression most often involves the use of selective serotonin reuptake inhibitors (SSRIs) which block 5-HTT, thereby increasing the amount of available serotonin for neural signaling [1,3]. Serotonin functioning also accounts for normal variation in temperament or personality [4,5,6,7]. Links have been reported in humans between variation in serotonergic signaling and scores on personality traits with high loadings for personality items related to impulsivity, aggression, and stress/anxiety [4,5,6,7]. For example, low serotonin activity typically relates to higher scores on Neuroticism, a personality dimension measured by the Revised NEO Personality Inventory (or NEO-PI-R). The dimension has six underlying facets: anxiety, angry hostility, depression, self-consciousness, impulsiveness, and vulnerability [4,8,9]. Other studies have reported a link with Harm Avoidance, a personality dimension that strongly correlates with Neuroticism [8], but is measured using a different personality assay, the Tridimensional Personality Questionnaire (TPQ) [9]. Individuals scoring high on Harm Avoidance tend to worry more, fear uncertainty more, be shyer with strangers, and are more susceptible to fatigue [9].

Several lines of evidence confirm the association between serotonin and anxiety-related personality dimensions. Serotonergic drugs treat psychopathological disorders with symptoms such as depression and anxiety that reflect high levels of Neuroticism [10,11]. Treatment with SSRIs decreases Neuroticism, which in turn mediates the effects of these drugs on the treatment of depressive symptoms [12]. Neurological evidence from PET (positron emission tomography) scans also shows that Neuroticism can be predicted based on the binding activity of serotonin to its receptor and/or transporter [4,13,14]. While Neuroticism is also estimated to have a significant heritable basis (15%) in humans [15], studies investigating the molecular genetic basis of the association between serotonin and Neuroticism are inconclusive and report rather small genetic effects [16]. Specific variants in the genes coding for the transporter and receptor have been shown to alter gene transcription, as well as protein structure and/or function, making individuals more prone to developing specific personality phenotypes [8,17,18,19,20]. More recent work has shown how external factors, such as early-life stress or trauma, can mediate these effects by influencing epigenetic processes, including DNA methylation [21]. In humans, epigenetic modifications of serotonergic genes contribute substantially to the pathogenicity of major depression and anxiety disorders [22,23]. For example, increased levels of methylation in the proximal promoter region of 5-HTT are associated with lower transporter expression and higher threat-related responsiveness of the amygdala [23], whereas lower methylation is correlated with better stress-adaptive reaction [22].

The role of epigenetic effects on the development of personality is gaining increasing interest but remains understudied in nonhuman primates. Chimpanzees offer an interesting comparative framework as they are, together with bonobos, genetically the most closely related living species to humans and thus show considerable overlap in the neural architecture supporting personality processes and physiology [24,25,26,27]. In chimpanzees, personality dimensions have also been quantified using identical methods to human personality research, yielding similar multidimensional personality models [28]. Comparative work in the two species can thus yield novel insights into the generalizability and functioning of serotonergic personality regulation across species. CpGs that show consistent cross-species effects would provide evidence for an evolutionarily stable regulatory system and can aid in the development of diagnostic tools or offer therapeutic targets for the treatment of serotonin-related psychiatric conditions in humans. Alternatively, if consistency across species is lacking, this potentially implies that neuroanatomical and brain gene expression differences in serotonergic systems might have functional and/or evolutionary significance.

In the current study, we investigated the link between blood CpG methylation levels of two key serotonergic genes (the gene coding for the serotonin transporter (*SLC6A4*) and the gene coding for its receptor subtype 1A (*HTR1A*)) and personality scores of captive chimpanzees. Chimpanzee personality data were assessed in a previous study using questionnaires [28]. This approach yielded six personality components: Reactivity/Undependability, Dominance, Openness, Extraversion, Agreeableness, and Methodical. Different from the human Neuroticism dimension, trait items related to anxiety, depression, and impulsive behavior do not fall within one dimension in chimpanzees, but are scattered among three dimensions: Reactivity, Dominance, and Extraversion (see Table 1 for item loadings for each dimension) [28]. We thus expected to find epigenetic serotonergic effects to primarily target those three dimensions.

Chimpanzee Dominance and Extraversion also showed significant heritability estimates (*h*^2^ = 0.195 and *h*^2^ = 0.381, respectively), which were dependent on early social rearing conditions, with mother-reared individuals showing higher heritability rates for both dimensions compared to chimpanzees reared by humans in a nursery [29]. Given that stress has been shown to impact serotonergic methylation in other species, with early life stress [21] or mild life stress [30] causing higher *SLC6A4* or *HTR1A* methylation levels in humans or rodents, respectively, we predicted that nursery-reared individuals might have higher serotonergic methylation levels compared to mother-reared individuals. Early social rearing conditions have previously been shown to mediate the association between dopaminergic methylation patterns and Extraversion in chimpanzees, but the effects were modest [31].

## 2. Materials and Methods

### 2.1. Subjects

All chimpanzees were housed at the National Center for Chimpanzee Care, which is part of the Michale E. Keeling Center for Comparative Medicine and Research (KCCMR), UT MD Anderson Cancer Center, Bastrop, TX. The guidelines from the American Psychological Association for the ethical treatment of animals in research were followed throughout this project. All chimpanzees were housed in social groups of 5–15 individuals with 24 h access to indoor/outdoor enclosures (except during cleaning). All groups were provided with bedding, climbing structures, and daily enrichment and foraging opportunities. All groups had similar feeding schedules, a commercially available primate diet with fresh produce, and ad libitum access to drinking water.

### 2.2. Personality

A chimpanzee-specific questionnaire was developed to determine personality traits in previous work and was validated using behavioral observations [28]. Personality data were obtained for 99 chimpanzees, 49 of which were included in this study as blood samples were available to determine corresponding methylation profiles (21 females, 29 males, mean age: 25 years, age range at time of personality sampling: 12–51 years). A total of 41 personality items were rated on a Likert scale from 1 to 7 (least to most descriptive) by a total of 17 staff members that had worked with the chimpanzees for at least 6 months and felt confident that they were familiar enough with them to produce accurate ratings (caregiver, trainer, enrichment technician, behavioral research coordinator, and colony manager). Every rated chimpanzee had lived at the facility for a minimum of two years prior to the personality study. Each expert was asked to rate as many chimpanzees as they felt confident rating, leading to each of the 17 raters scoring on average 72 chimpanzees (ranging from 9 to 99). Interrater reliabilities were analyzed using intraclass correlation coefficients and were found to be high (mean ICC = 0.61; range 0.35–0.85) for all adjectives except for “predictable” (ICC= −0.05), which was therefore excluded from further analysis (for detailed results of ICC analysis, see [28]).

Six personality domains were revealed using principal component analysis on the means across raters: Reactivity/Undependability, Dominance, Extraversion, Openness, Agreeableness, and Methodical (see Table 1 for item loadings for each factor and Supplementary Table S1 for detailed adjective scores). The validity of the structure of these six factors was then further evaluated independently by three experts in chimpanzee behavior who worked as behavioral researchers with the chimpanzees for at least three years. These experts did not participate in the initial rating process and examined the rating construct for convergent and predictive validity (for more details see [28]). Five out of six dimensions show strong evidence for convergent and predictive validity, meaning they are consistent among raters and among studies, and show expected correlations with independently collected quantitative behavioral observations [28,32,33,34]. Three out of six dimensions (Agreeableness, Dominance, Reactivity/Undependability) showed significant stability up to ten years between ratings, with Dominance showing the strongest stability [35]. Given that the personality dimension “Methodical” tended to differ between studies, and showed poor construct validity and low stability over time, we excluded it here. Construct validity of the personality questionnaire was carried out for individuals of all age ranges included in this study [28,35].

### 2.3. DNA Extraction and Methylation

Blood samples were used to extract DNA for methylation analysis for 49 chimpanzees with available personality profiles and known early social rearing conditions. Blood sampling was carried out when chimpanzees were between 12 and 51 years old, and the time between blood sampling and collection of personality ratings was on average 2.11 years (SD = 3.96). Blood samples were taken in anaesthetized animals, stored in EDTA, and frozen at −80 °C. Extractions of genomic DNA were performed with the automated QiaCube (Qiagen, Germantown, MD, USA) using the QIAampDNA Mini Kit for 200 μL of whole blood. Extracted DNA samples were brought to a concentration of ~70 ng/μL and concentrations were confirmed with a Nanodrop 2000 (Thermo-Fisher Scientific, Waltham, MA, USA) spectrophotometer. Next, samples underwent bisulfite conversion and were then analyzed using the Illumina Infinium Methylation EPIC array at the Yale Center for Genome Analysis (New Haven, CT, USA). This array was designed for measuring methylation levels at over 850,000 CpG sites in the human genome. Therefore, for the purpose of using it in chimpanzees, we limited our dataset to CpG sites targeted by probes that map to the chimpanzee (panTro2.1.4) genome with 0–2 mismatch, with no mismatches within 5 bp of the target CpG, and with no SNPs known in chimpanzees following [36]. We also filtered out probes with spectral intensities not significantly different from background levels, that do not target CpG dinucleotides, that are on the sex chromosomes, and for which fewer than 3 beads were counted for 5% or more of the samples with the ChAMP v2.18.3 R package [37]. Because blood tissue is a composite of multiple cell types with proportions that vary over time and across individuals, we corrected the methylation data for variation in cell type composition using the refbase function in the ChAMP package [37].

### 2.4. Statistical Analysis

#### 2.4.1. Methylation vs. Personality

All statistical analyses were performed in R (www.r-project.org, version 3.3.2, R Core Team, Vienna, Austria). To test for *HTR1A* and *SLC6A4* methylation effects on personality, we ran linear models using the lm function in the lme4 package in R [38]. Each personality factor was tested as an outcome variable in a separate model. As for fixed effects, we included sex and methylation scores at individual CpG sites for each gene [21]. The age at which blood was collected and individual relatedness coefficients were entered as covariates. Relatedness coefficients were calculated using the degree of relatedness of each individual to all other individuals in the colony and were extracted based on the entire pedigree using the kinship2 package in R (https://cran.r-project.org/package=kinship2, accessed on 27 November 2018). Model selection was performed using the Akaike Information Criterion (AIC). We examined diagnostic plots (residuals vs. leverage, QQ plots, etc.) and Shapiro–Wilk tests to confirm the assumptions of linear models. As we included individual CpG sites, we employed a *p*-value correction using a false discovery rate (FDR) correction of 5% for each personality dimension to account for multiple testing [39]. To allow for cross-study comparison of our results, we also included a principal component approach sometimes used in the literature that reduces model complexity by clustering the CpGs that are present for each gene (N*_HTR1A_* = 22, N*_SLC6A4_* = 26) in fewer overarching dimensions (Supplementary Tables S2 and S3). None of the associations between personality dimensions and PCA scores were significant after FDR correction (Supplementary Table S4).

#### 2.4.2. Rearing vs. Methylation

Finally, differences in methylation scores were tested between individuals with different early social rearing backgrounds for those CpGs that showed a significant association with personality. Mother-reared individuals were tested against individuals that were raised by human care staff in a nursery setting. These models were run separately from the models above, given that we excluded three wild-born individuals from this analysis. This was done because a sample size of three does not allow for meaningful statistical comparison to the other two rearing categories, and because the background of wild-born individuals is often complex as they were partially mother- and human-reared, depending on how old they were when they entered into captivity. For the remaining 13 nursery-reared and 34 mother-reared chimpanzees in our sample, we treated individual CpG methylation scores as outcome variables and rearing backgrounds as fixed effects. A correction for age, sex, and relatedness remains in the model, as similarly described for the linear models above. Both mother- and nursery-reared chimpanzees had access to outdoor enclosures and showed minimal variance in environmental factors outside of rearing conditions. Both were reared in similarly sized social groups with changing composition over the years due to individuals being introduced, passing away, or being moved between social groups for breeding purposes. They also experienced similar feeding routines and access to water, enrichment, bedding, and climbing opportunities.

## 3. Results

### 3.1. HTR1A and SLC6A4 Methylation Sites

We identified 21 *HTR1A*-specific CpG sites and 27 *SLC6A4*-specific CpG sites and confirmed their presence in the chimpanzee genome. Coordinates and IDs of individual CpG sites in the human and chimpanzee genome are shown in Table 2.

### 3.2. Associations between CpG Methylation Scores and Personality

**Figure 1:**
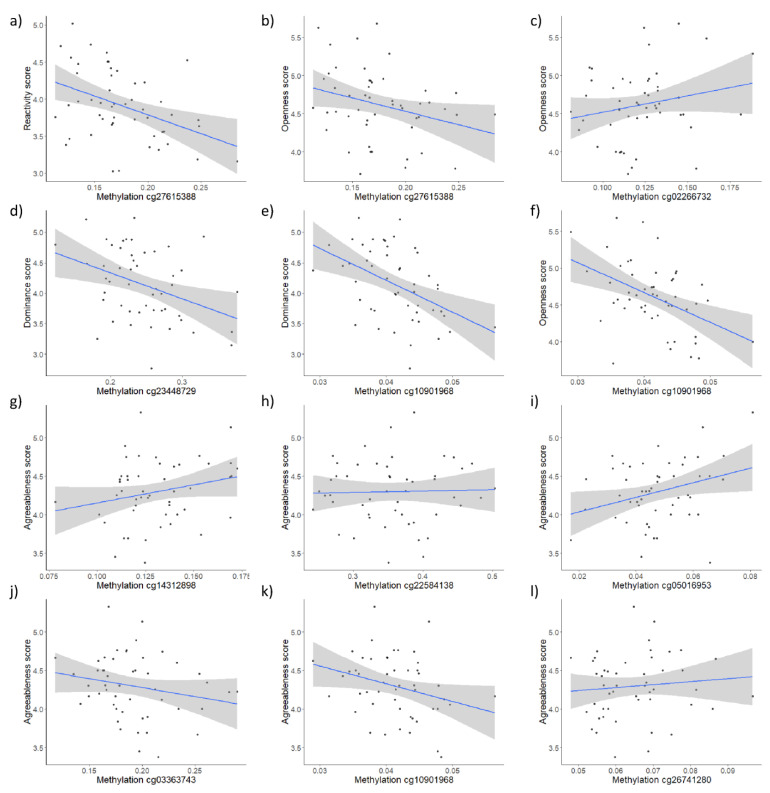
Significant associations between individual CpG methylation scores and personality domains for HTR1A (**a**–**d**) and SLC6A4 (**e**–**l**).

Methylation levels of nine CpG sites (three for *HTR1A* and six for *SLC6A4*) were significantly associated with four personality traits (Dominance, Agreeableness, Reactivity, and Openness) after FDR correction for multiple testing (Table 3, Figure 1). Higher methylation of SLC6A4 probe cg10901968 predicted lower Dominance (p_adj_ = 0.005), Agreeableness (p_adj_ = 0.003), and Openness (p_adj_ = 0.022) scores. Similarly, the higher methylation of *HTR1A* probe cg23448729 was related to lower Dominance scores (p_adj_ = 0.021) while higher scores for *HTR1A* probe cg27615388 predicted lower scores for Reactivity (p_adj_ = 0.001) and Openness (p_adj_ = 0.038). Conversely, higher scores for *HTR1A* probe cg02266732 predicted higher scores for Openness (p_adj_ = 0.038). Finally, Agreeableness scores were further significantly associated with scores for five more *SLC6A4* probes, four of which showed positive associations (cg14312898: padj = 0.006; cg22584138: p_adj_ = 0.038; cg05016953: p_adj_ = 0.003; cg26741280: p_adj_ = 0.012), with one showing a negative association (cg26741280: p_adj_ = 0.034). For an overview of remaining associations between individual CpGs and personality domains, see Supplementary Tables S5–S9.

### 3.3. Influence of Rearing on Methylation

**Figure 2:**
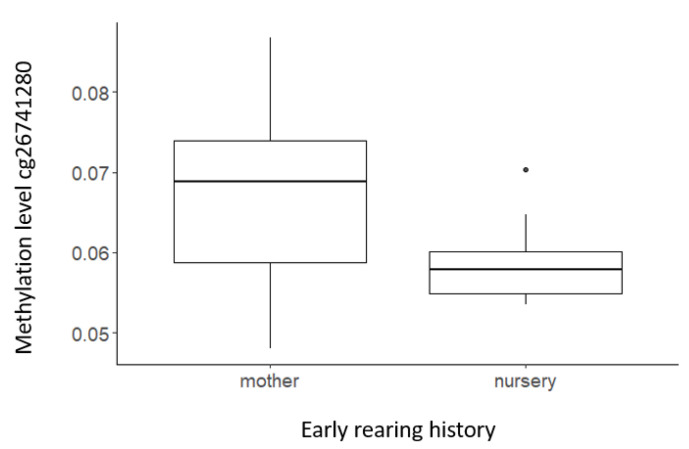
Impact of early rearing history on methylation scores for cg26741280.

Methylation levels of only one out of the nine individual CpG probes that showed significant associations with personality domains were significantly influenced by early rearing history (Table 4), where methylation levels of mother-reared individuals were higher for *SLC6A4* probe cg26741280 than for nursery-reared ones (t = −7.345, df = 45, p_adj_ = 0.001; Figure 2).

## 4. Discussion

In this study, we investigated the association between epigenetic modification of two key serotonergic genes (*HTR1A* and *SLC6A4*) and the expression of personality in captive chimpanzees. Methylation levels at different CpG sites in both genes showed significant associations with four personality dimensions: Dominance, Reactivity, Agreeableness, and Openness.

While 48 CpG sites were present in both genes combined, only nine showed significant associations with personality, three of which were located on *HTR1A*, with the remaining six on *SLC6A4*. For *HTR1A*, these three CpGs showed associations with three different personality dimensions: Dominance, Reactivity, and Openness. Higher methylation of cg02266732, a CpG located in the CpG island in the 200 bp region upstream of the transcription start site, was associated with higher scores for Openness, indicating that those chimpanzees were rated higher on items such as Human oriented, Inquisitive/Curious, Inventive, Intelligent, Affectionate/Friendly, and Persistent. DNA methylation of CpGs located in or near the promoter region generally results in reduced expression of the gene [40], which indicates that in individuals with higher methylation levels at this site, fewer HTR1A receptors are available.

As HTR1A typically has an inhibitory effect on serotonin production [41], having fewer of them would result in increased serotonergic signaling, which explains the higher levels of explorative and social personality profiles in these chimpanzees through serotonin-induced anxiety relief. For the other two CpG sites, the association between methylation and behavior was reversed. Higher methylation scores for cg23448729, located in the *HTR1A* exon, were associated with lower Dominance scores. This means that individuals would be scored as more anxious and cautious, while scoring lower for bold and independent. Similarly, higher methylation of cg27615388, also located in exon 1, predicted lower Dominance scores, but also lower Reactivity and Openness scores, meaning individuals would be more irritable, aggressive, or socially inept and less explorative and affectionate. For both CpGs, these results are in line with reduced serotonergic activity and thus likely higher levels of inhibitory *HTR1A* transcription. The fact that the directionality of the association between serotonergic methylation and behavior changes is dependent on the location of the significant CpG loci is in line with previous findings where positive associations were typically found between methylation and gene expression in probes located in the gene body, whereas negative ones were found for probes closer to the transcription start site [42].

For the serotonin transporter gene, methylation scores for six CpGs were found to be associated with three different personality dimensions. Higher levels of methylation of cg10901968, a CpG site located within the CpG island in the 200 bp region upstream of the transcription start site of the gene, was associated with lower Dominance, Agreeableness, and Openness scores, indicating that these individuals would overall behave more anxious and cautious, less considerate and protective of other chimpanzees, and less explorative, and affectionate. These results are in line with our expectations based on the literature, where in general, higher levels of *SLC6A4* methylation result in lower *SLC6A4* expression, which results in higher risk for depression and anxiety and reduced behavioral stress reactivity. The higher methylation levels of the remaining five *SLC6A4* CpGs were associated with either higher (cg14312898, cg26741280, cg05016953, cg22584138) or lower Agreeableness scores (cg03363743), with no clear pattern for the impact of the location of the CpG inside or outside of the CpG island or gene body.

The Agreeableness dimension only has two item loadings (protective and considerate); chimpanzees scoring highly here are considered to be individuals that show higher levels of concern for other chimpanzees, intervene more often to prevent harm or annoyance coming to them, and console them when in distress to provide reassurance. While this dimension might reflect empathetic capacity and/or prosociality in chimpanzees, this requires confirmation with additional behavioral testing. Data from coded behavioral observations do show that individuals scoring highly on Agreeableness show more affiliative behaviors and were less likely to displace others and solicit other chimpanzees for assistance during fights [28]. While the socio-negative effects of low serotonin are more often emphasized in the literature due to its role in anxiety disorders and depression, its reversed associations with socio-positive behaviors are not unexpected. Although reports are scarcer, studies in both animals and humans have documented links between higher serotonin activity and higher levels of prosocial and affiliative behaviors, and suggest that serotonin might actually function to promote an individual’s potential for self-control of emotional reactivity and increase its aversion to the harming of others [43]. Previously, we showed that in chimpanzees, a genetic mutation causing an amino acid change in *HTR1A* (Pro248Gln) is associated with both higher levels of socio-negative behaviors (anxiety and aggression) and lower levels of two socio-positive behaviors (grooming and proximity) [44].

Interestingly, no significant associations were found between the *HTR1A* polymorphism and personality dimension scores in this larger cohort of chimpanzees (N = 214) that included all chimpanzees from the current study [44], confirming the claims that serotonergic genetic effects on personality are modest [16]. Epigenetic effects thus appear to have a bigger impact on chimpanzee personality than genetic ones, but this requires further testing in additional chimpanzee populations. Previous work did report potential genotype effects of a repeat polymorphism in the promoter region of *SLC6A4* known as 5-HTTLPR (serotonin transporter-linked promoter region) on methylation levels of specific CpG sites (cg18584905, cg10901968, cg25725890, cg22584138, cg05951817; see [45,46]). Of these CpGs, cg22584138 and cg10901968 showed significant positive associations with chimpanzee personality in our study, which could thus be mediated through underlying 5-HTTLPR genotype differences. While the investigation of genotype by methylation interaction effects on behavioral profiles would offer promising avenues for future work, a larger dataset would be needed to obtain sufficient power to disentangle these effects. Developing large-scale studies in great apes is challenging as they require both blood and/or post-mortem tissue samples, which are collected opportunistically, and matching behavioral profiles.

Despite sample size limitations, our study yields important clues regarding the reproducibility and generalizability of human findings to other species. Especially for *SLC6A4*, where methods used across studies are less heterogeneous [46], comparisons can be made for CpG site-specific phenotype associations. These show that at least for two of the CpGs associated with chimpanzee Agreeableness (cg05016953 and cg22584138), functional comparative validity with human studies is present. Human studies report links for these CpGs with differential 5-HTT expression patterns [47] and with responsiveness to antidepressant treatment (cg05016953) [48]. This indicates that methylation levels of these CpG sites have robust, evolutionarily conserved effects on 5-HTT functioning. Unfortunately, studies investigating links between individual serotonergic methylation and personality so far tend to focus on personality disorders (antisocial personality disorder, borderline personality disorder) or rather report links with behavioral tendencies (aggressiveness, antisocial behavior) instead of personality dimensions that rely on a similar construct to ours (cf. the Big Five) (for a review, see [2]). More studies are thus needed to confirm the cross-species validity of CpG effects on personality.

The results do clearly support a role of serotonin methylation in chimpanzee personality regulation that differs from that of dopamine. In the same sample of chimpanzees, methylation levels of the dopamine receptor type D2 (*DRD2*) previously showed strong associations with Extraversion and Openness, two personality dimensions that reflect a tendency to actively explore or engage with novelty [31]. While a modest link with Openness was present in our study for three out of 48 serotonergic CpG sites, no associations were found with Extraversion, while scores for Extraversion were significantly associated with methylation levels of 13 out of 16 *DRD2* CpG sites. Although the serotonin system is more involved in self-regulation and emotional stability and targets the limbic system of the brain, dopamine stimulates exploration-related personality traits through reward-center activation [49]. Thus, both neurotransmitters have closely intertwined functions, but methylation patterns in the two systems regulate personality in rather distinct ways.

Finally, we investigated whether atypical early social conditions in nursery-reared individuals accounted for some of the variation in serotonergic methylation patterns in our population of chimpanzees. For those nine CpGs that showed associations with chimpanzee personality, only one was differentially methylated between nursery-reared versus mother-reared chimpanzees. This CpG site, cg26741280, was located in a CpG island, close to the transcription start site of the serotonin transporter gene (*SLC6A4*), where nursery-reared individuals showed lower methylation levels than mother-reared individuals. These results are in contrast to the general consensus that childhood adversity leads to overall higher levels of *SLC6A4* methylation in humans (for a review, see [21]) and rhesus macaques [50], but align with findings where promoter-specific CpG sites were undermethylated in individuals in high-stress environments [51]. The contrast between these studies is likely explained by the heterogeneity in the methods that were used. As evidenced by our results above, the location of the CpG sites is important in determining the direction of the effect, something that average methylation scores across genes do not take into account [42]. It is thus possible that nursery-reared individuals experience reduced long-term stress resistance compared to mother-reared ones, which is associated with decreased methylation of promoter CpG cg26741280 and higher *SLC6A4* transcription and results in lower Agreeableness. The effects of rearing are nonetheless modest, as evidenced by only one CpG being affected by rearing background; functional assays are needed to investigate to what extent cg26741280 methylation levels impact *SLC6A4* transcription in chimpanzees. The modest effects of the atypical early-rearing conditions on methylation are in line with findings for *DRD2* in chimpanzees [31] but contrast with findings in humans and require further investigation. One explanation could be that the impact of nursery rearing on chimpanzees under attentive care from humans is less traumatic and therefore only has modest effects on neural circuitry, a claim that is supported by differences in personality scores for adult chimpanzees from both background conditions. Nursery-reared individuals do not necessarily differ in personality scores for traits related to anxiety or stress (Dominance, Impulsivity) from mother-reared ones, but rather score differently on traits related to affiliative, friendly, human-oriented, and intelligent behavior (Agreeableness, Intellect) [29]. Alternatively, it is possible that factors other than rearing conditions play a more prominent role in determining adult serotonergic methylation patterns in chimpanzees. In humans, for example, links have been documented with prenatal infections, childhood exposure to greenness in the environment, and medicine use [52,53]. In chimpanzees, future studies could therefore investigate what additional factors have an impact on methylation status. Low dominance rank, levels of aggression received, low levels of affiliation received, changing group dynamics, transfers to novel social groups, medical history, and prenatal and maternal effects may impact epigenetic modification, but relatively large sample sizes are required for accurate investigation.

## 5. Conclusions

In conclusion, our results confirm an evolutionarily stable role of serotonergic methylation in reducing anxiety- (Dominance) and aggression- (Reactivity/Undependability) related personality aspects while simultaneously promoting prosocial (Agreeableness) and exploratory (Openness) personalities. They also show that methylation effects on chimpanzee personality were CpG-specific and heavily dependent on the location of the CpG [40,42]. This highlights the importance of investigating methylation patterns at the CpG site level as opposed to using average gene methylation scores or technologies such as mass spectrometry that do not allow CpG-specific methylation resolution [21]. These results offer an important basis for future hypothesis-driven work that can target specific CpGs and investigate their role in regulating neuroanatomical and/or behavioral phenotypes in other populations and species.

## Figures and Tables

**Table 1:** Adjective loadings for varimax-rotated chimpanzee personality traits.

Trait	Adjectives/Behaviors
Reactivity/ Undependability	+ Irritable + Temperamental/moody + Deceptive + Impulsive + Defiant + Mischievous + Jealous + Manipulative + Stingy + Bullying + Aggressive + Eccentric + Socially inept + Excitable + Autistic − Calm
Dominance	+Bold + Relaxed + Dominant − Fearful − Timid − Cautious − Dependent − Anxious
Extraversion	+Active + Playful + Sexual + Affiliative − Solitary − Depressed
Openness	+ Human oriented + Inquisitive/curious + Inventive + Intelligent + Affectionate/ Friendly + Persistent
Agreeableness	+ Protective + Considerate
Methodical	+ Self-caring + Methodical

**Table 2:** 48 Chimpanzee serotonergic CpG sites identified and included in this study: 21 in the serotonin receptor gene 1A (*HTR1A*) and 27 in the serotonin transporter gene (*SLC6A4*).

Gene	CpG ID	Functional Region	Location	CHR	Coordinate hg19
*HTR1A*	cg11432303	TSS1500	shore	5	63,259,058
	cg20598238	TSS1500	shore	5	63,258,783
	cg08764163	TSS1500	shore	5	63,258,768
	cg27420687	TSS1500	shore	5	63,258,592
	cg16807523	TSS1500	shore	5	63,258,434
	cg10198270	TSS200	shore	5	63,258,311
	cg13666507	TSS1500	shore	5	63,257,941
	cg07839533	TSS1500	island	5	63,257,885
	cg15092168	TSS1500	island	5	63,257,873
	cg11615755	TSS1500	island	5	63,257,867
	cg09698471	TSS1500	island	5	63,257,847
	cg08259925	TSS1500	island	5	63,257,813
	cg16280141	TSS1500	island	5	63,257,753
	**cg02266732**	**TSS200**	**island**	**5**	**63,257,710**
	cg04694812	TSS200	island	5	63,257,554
	cg04427003	1stExon	island	5	63,257,499
	cg17386123	1stExon	island	5	63,257,353
	**cg27615388**	**1stExon**	**island**	**5**	**63,257,092**
	cg04799838	1stExon	island	5	63,256,926
	cg10588470	1stExon	island	5	63,256,619
	**cg23448729**	**1stExon**	**shore**	**5**	**63,256,285**
*SLC6A4*	cg12074493	TSS1500	shore	17	28,564,117
	cg06841846	TSS1500	shore	17	28,564,094
	**cg14312898**	**TSS1500**	**shore**	**17**	**28,563,979**
	cg03829016	TSS1500	shore	17	28,563,859
	cg18584905	TSS1500	shore	17	28,563,300
	**cg10901968**	**TSS200**	**island**	**17**	**28,563,108**
	**cg26741280**	**TSS200**	**island**	**17**	**28,563,089**
	cg25725890	TSS200	island	17	28,563,054
	**cg05016953**	**1stExon**	**island**	**17**	**28,562,813**
	cg06373684	1stExon	island	17	28,562,751
	cg26438554	1stExon	island	17	28,562,733
	cg14692377	1stExon	island	17	28,562,685
	**cg03363743**	**5’UTR**	**island**	**17**	**28,562,474**
	**cg22584138**	**5’UTR**	**shore**	**17**	**28,562,220**
	cg05951817	5’UTR	shore	17	28,562,142
	cg00386645	5’UTR	shore	17	28,560,965
	cg10241426	5’UTR	shelf	17	28,558,934
	cg16647683	5’UTR	opensea	17	28,558,098
	cg01991100	5’UTR	opensea	17	28,555,935
	cg09921370	5’UTR	opensea	17	28,555,315
	cg01330016	5’UTR	opensea	17	28,549,806
	cg10146136	Gene body	opensea	17	28,547,550
	cg27427014	Gene body	opensea	17	28,539,980
	cg08743901	Gene body	opensea	17	28,535,556
	cg06961290	Gene body	opensea	17	28,535,040
	cg20209182	Gene body	opensea	17	28,530,849
	cg20592995	3’UTR	opensea	17	28,524,160

CHR—chromosome number; 5′ UTR—5 end untranslated region; TSS1500—1500 bp region upstream of transcription site; TSS200—200 bp region upstream of transcription site; 3′ UTR—3 end untranslated region. Boldface indicates that CpGs are significantly associated with chimpanzee personality in this study.

**Table 3:** Model statistics for *HTR1A* and *SLC6A4* individual CpG scores and their significant associations with personality dimensions in chimpanzees.

Personality	Gene	Cg Probe	Est	SE	t	*p*	p_adj_	Sign.
Dominance	*HTR1A*	cg23448729	−6.129	1.652	−3.710	0.001	0.021	*
	*SLC6A4*	cg10901968	−59.575	14.500	−4.109	0.000	0.005	**
Agreeableness	*SLC6A4*	cg14312898	7.521	2.051	3.667	0.001	0.006	**
		cg22584138	2.375	0.859	2.766	0.008	0.038	*
		cg05016953	14.287	3.562	4.011	0.000	0.003	**
		cg03363743	−4.568	1.587	−2.879	0.006	0.034	*
		cg10901968	−37.428	9.303	−4.023	0.000	0.003	**
		cg26741280	15.793	4.740	3.331	0.002	0.012	*
Reactivity	*HTR1A*	cg27615388	−7.345	1.646	−4.463	0.000	0.001	**
Openness	*HTR1A*	cg02266732	8.661	2.820	3.072	0.004	0.038	*
		cg27615388	−5.184	1.627	−3.187	0.003	0.038	*
	*SLC6A4*	cg10901968	−38.955	10.859	−3.587	0.001	0.022	*

* indicates *p* < 0.05; ** indicates *p* < 0.01 (after FDR correction for multiple testing).

**Table 4:** Model statistics for rearing effects on methylation rates of personality-related CpG sites.

Gene	Cg probe	Est	SE	t	*p*	Sign.
*HTR1A*	cg23448729	−0.018	0.017	−1.103	0.276	
	cg27615388	−0.006	0.013	−0.473	0.639	
	cg02266732	0.006	0.008	0.715	0.479	
*SLC6A4*	cg14312898	0.007	0.007	0.932	0.357	
	cg22584138	0.004	0.023	0.160	0.874	
	cg05016953	0.004	0.004	0.999	0.323	
	cg03363743	0.009	0.012	0.787	0.436	
	cg10901968	0.000	0.002	−0.256	0.799	
	cg26741280	−0.008	0.003	−2.870	0.006	**

Estimates are for nursery-reared individuals in comparison to mother-reared chimpanzees. ** indicates *p* < 0.01.

## Data Availability

Data are available in Supplementary Tables S10 and S11.

## Supplementary Materials

The following supporting information can be downloaded at https://www.mdpi.com/article/10.3390/biology11111673/s1. Table S1: Factor loadings of chimpanzee personality traits for six varimax-rotated factors; Table S2: *HTR1A* CpG factor item loadings for varimax-rotated factors; Table S3: *SLC6A4* CpG factor item loadings for varimax-rotated factors; Table S4: Model statistics for *HTR1A* and *SLC6A4* CpG composite measure scores and their association with personality dimensions in chimpanzees; Table S5: Model statistics for *HTR1A* and *SLC6A4* individual CpG methylation scores and their association with Dominance scores in chimpanzees; Table S6: Model statistics for *HTR1A* and *SLC6A4* individual CpG methylation scores and their association with Agreeableness scores in chimpanzees; Table S7: Model statistics for *HTR1A* and *SLC6A4* individual CpG scores and their association with Openness scores in chimpanzees; Table S8: Model statistics for *HTR1A* and *SLC6A4* individual CpG scores and their association with Reactivity scores in chimpanzees; Table S9: Model statistics for *HTR1A* and *SLC6A4* individual CpG scores and their association with Extraversion scores in chimpanzees; Table S10: Dataset with individual name, sex, age at personality rating, age at blood, relatedness coefficient, personality dimension scores, Principal Compont scores for each component for HTT or HTR and individual CpG site scores; Table S11: Dataset with individual name, sex, age at personality rating, age at blood sampling, relatedness coefficient, early social rearing conditions, personality dimension scores, Principal Compont scores for each component for HTT or HTR and individual CpG site scores.
