# Supplementary material for: The Role of Serotonergic Gene Methylation in Regulating Anxiety-Related Personality Traits in Chimpanzees
Source: Biology (Basel). 2022 Nov 17;11(11):1673. doi: 10.3390/biology11111673 (PMC9687614; doi:10.3390/biology11111673)
Supplement: Supplementary file 1 [file biology-11-01673-s001.zip › Supplementary information-1.pdf]

Table S1. : Factor loadings of chimpanzee personality traits on six varimax-rotated factors.

Trait	Reactivity/ Undependability	Dominance	Extraversion	Openness	Agreeableness	Methodical
Irritable	**0.87**	-0.09	-0.05	-0.09	-0.13	0.15
Temp./moody	**0.85**	-0.01	0.04	-0.02	-0.08	0.18
Deceptive	**0.79**	-0.13	0.12	0.18	0.18	0.05
Impulsive	**0.77**	-0.04	0.28	0.27	0.00	-0.12
Defiant	**0.74**	-0.06	0.31	0.22	-0.03	-0.10
Mischievous	**0.73**	0.07	0.41	0.33	-0.01	-0.13
Jealous	**0.70**	-0.07	0.35	0.40	0.02	0.05
Manipulative	**0.68**	-0.35	0.14	0.18	0.25	0.09
Stingy	**0.68**	-0.49	0.07	-0.06	0.23	0.03
Bullying	**0.68**	-0.56	0.25	-0.07	0.06	0.03
Aggressive	**0.66**	-0.42	0.41	-0.12	-0.06	0.14
Eccentric	**0.62**	0.13	-0.22	0.36	-0.04	-0.14
Socially-inept	**0.58**	0.36	0.02	0.07	-0.37	-0.06
Calm	**-0.57**	-0.06	-0.50	0.02	0.37	-0.10
Excitable	**0.56**	-0.08	0.49	0.19	-0.22	0.09
Autistic	**0.42**	0.36	-0.22	0.05	-0.03	-0.28
Fearful	0.03	**-0.88**	-0.11	0.05	-0.12	-0.01
Timid	-0.14	**-0.84**	-0.27	-0.23	-0.09	-0.04
Cautious	-0.23	**-0.81**	-0.11	-0.13	0.07	-0.01
Dominant	0.40	**0.78**	0.16	-0.03	0.18	0.13
Dependent	-0.02	**-0.76**	0.21	0.01	0.30	-0.15
Anxious	0.32	**-0.75**	0.28	-0.07	-0.05	0.20
Bold	0.53	**0.61**	0.35	0.29	0.12	0.05
Relaxed	-0.44	**0.48**	-0.46	0.05	0.31	-0.15
Solitary	-0.18	0.16	**-0.77**	-0.18	-0.29	0.08
Depressed	0.03	0.32	**-0.76**	-0.13	0.02	0.01
Active	0.26	0.09	**0.72**	0.47	-0.10	0.12
Playful	0.20	0.06	**0.67**	0.58	-0.06	-0.09
Sexual	0.29	-0.07	**0.65**	0.01	0.30	0.22
Affiliative	0.09	0.09	**0.53**	0.43	0.49	0.02
Human oriented	0.08	-0.14	-0.02	**0.83**	0.01	0.05
Inq./Curious	0.26	-0.01	0.30	**0.80**	-0.03	0.00
Inventive	0.28	0.03	0.20	**0.76**	0.12	0.14
Intelligent	-0.01	-0.19	0.09	**0.70**	-0.08	0.50
Aff./Friendly	-0.28	-0.03	0.27	**0.61**	0.41	-0.11
Persistent	0.46	-0.34	0.10	**0.54**	0.11	0.15
Protective	0.15	-0.20	0.06	-0.08	**0.78**	0.15
Considerate	-0.44	0.16	-0.01	0.25	**0.63**	0.14
Self-caring	0.05	0.06	0.20	0.31	0.32	**0.55**
Methodical	0.16	-0.44	-0.26	0.30	0.33	**0.54**

Boldface indicates the items with loadings >|0.40| that load onto the factor

PCA approach

PCAs were done on individual CpG scores per gene, using varimax rotation with Kaiser normalization and the number of dimensions to extract was determined based on parallel analysis (53). Coefficients of correlation for methylation scores >|0.5| were considered high and therefore salient and kept for further analysis, resulting in two six component models (Table S1 and S2). For both PCAs, inter-variable correlations were sufficiently high (Bartlett's test of sphericity$_{HTR1A}$: χ^2 =526.29, df =210, p < 0.001; Bartlett's test of sphericity$_{SLC6A4}$: χ^2 =711.69, df =300, p < 0.001) and sampling adequacy was high (KMO$_{HTR1A}$ = 0.683; KMO$_{SLC6A4}$ = 0.686). For *HTR1A*, all CpG sites showed salient loadings on at least one PC and were therefore included in the analysis but for *SLC6A4* two CpGs did not load (cg06961290 and cg20592995) and were therefore dropped from further analysis.

Table S2. *HTR1A* CpG factor item loadings on varimax-rotated factors.

CpG ID	PC1	PC2	PC3	PC4	PC5	PC6	*h*2
cg04799838	**0.857**	-0.078	-0.049	0.066	0.093	0.015	0.756
cg10588470	**0.751**	0.044	0.315	0.212	-0.017	-0.128	0.728
cg23448729	**0.688**	0.399	0.329	0.189	0.104	-0.054	0.790
cg04427003	**0.687**	-0.083	0.091	0.047	-0.006	0.348	0.610
cg27615388	**0.677**	0.198	0.188	0.044	0.370	0.340	0.788
cg04694812	**0.530**	0.010	-0.053	-0.434	0.399	0.098	0.641
cg20598238	-0.128	**0.905**	-0.021	0.005	0.057	0.005	0.840
cg08764163	0.086	**0.722**	0.022	0.400	-0.032	0.138	0.709
cg11615755	0.250	**0.543**	0.236	0.152	-0.067	0.207	0.483
cg16807523	0.111	**0.541**	0.226	0.019	0.429	0.024	0.541
cg15092168	0.005	0.052	**0.835**	0.060	0.116	0.153	0.740
cg08259925	0.279	0.194	**0.75**	-0.006	-0.039	0.051	0.682
cg27420687	0.082	0.230	-0.064	**0.836**	0.014	0.218	0.811
cg13666507	0.238	-0.026	0.063	**0.769**	-0.139	-0.179	0.704
cg10198270	-0.003	0.278	0.234	**0.598**	0.432	0.191	0.713
cg16280141	0.233	-0.003	-0.046	-0.098	**0.738**	0.094	0.620
cg17386123	0.005	0.553	0.203	-0.192	**0.584**	0.014	0.725
cg07839533	0.132	-0.137	0.515	0.188	**0.554**	0.051	0.647
cg11432303	-0.322	0.474	-0.075	0.229	**0.53**	0.303	0.759
cg02266732	0.093	0.112	0.127	0.112	0.168	**0.855**	0.810
cg09698471	0.285	0.253	0.544	-0.147	0.056	**0.609**	0.837
Eigenvalue	5.888	2.933	2.226	1.472	1.314	1.101	
% variance explained	28.039	13.966	10.6	7.007	6.258	5.244	

Table S3. SLC6A4 CpG factor item loadings on varimax-rotated factors.

CpG ID	PC1	PC2	PC3	PC4	PC5	PC6	h^2
cg10146136	**0.839**	0.129	0.170	-0.129	0.040	-0.115	0.780
cg00386645	**0.766**	0.117	0.121	0.073	-0.156	0.164	0.671
cg06841846	**-0.666**	0.209	-0.009	0.224	0.364	0.050	0.672
cg10241426	**0.664**	0.161	0.418	0.071	-0.267	-0.008	0.718
cg14312898	**0.585**	0.533	0.138	0.054	0.007	0.092	0.657
cg01991100	**0.577**	0.460	0.459	0.195	-0.005	0.033	0.795
cg16647683	**0.472**	0.269	0.407	0.042	-0.358	-0.113	0.603
cg06373684	0.001	**0.854**	-0.095	0.178	0.029	-0.149	0.793
cg08743901	0.098	**0.772**	0.066	-0.240	0.017	-0.103	0.679
cg26438554	0.168	**0.733**	-0.154	0.369	0.036	0.014	0.726
cg03829016	0.013	**0.728**	-0.118	0.089	0.005	0.438	0.743
cg05016953	-0.157	-0.071	**-0.817**	0.170	-0.124	0.205	0.783
cg14692377	0.049	0.233	**-0.788**	0.235	0.188	-0.024	0.769
cg25725890	-0.223	0.019	**-0.666**	-0.254	0.172	0.118	0.601
cg01330016	0.335	-0.122	**0.601**	-0.080	-0.145	0.159	0.541
cg20209182	0.331	0.526	**0.534**	0.188	-0.129	0.129	0.741
cg05951817	-0.019	0.157	0.016	**0.910**	-0.122	-0.020	0.869
cg03363743	-0.121	0.043	-0.169	**0.869**	0.069	-0.219	0.853
cg22584138	-0.011	0.036	-0.015	**0.857**	0.047	0.073	0.743
cg27427014	0.405	0.198	0.188	**0.410**	0.285	-0.039	0.489
cg10901968	-0.019	0.014	-0.207	0.031	**0.860**	0.066	0.788
cg26741280	-0.233	0.005	-0.075	-0.051	**0.754**	-0.129	0.648
cg12074493	-0.497	0.051	0.003	0.178	**0.514**	0.257	0.611
cg18584905	-0.077	-0.099	-0.158	-0.248	0.105	**0.835**	0.811
cg09921370	0.178	0.400	0.171	0.216	-0.370	**0.528**	0.683
Eigenvalue	6.630	4.154	2.386	1.829	1.505	1.263	
% variance explained	26.519	16.615	9.543	7.316	6.018	5.051	

Two associations were found between methylation components and personality dimensions that only approached significance after FDR correction (Table S3). Scores on Dominance showed a trend for a negative association with methylation scores on *SLC6A4* PC5 ($t = -2.830$, $df = 45$, $p_{adj} = 0.084$), while scores on Agreeableness approached a positive association with methylation scores of *HTR1A* PC2 ($t = 0.148$, $df = 45$, $p_{adj} = 0.060$). For associations between the remaining components and personality dimensions see Table S3.

Table S4: Model statistics for *HTR1A* and *SLC6A4* CpG composite measure scores and their association with personality dimensions in chimpanzees

		Est	SE	t	p	p adj
Dominance	HTR_PC1	-0.268	0.126	-2.133	0.040	0.160
	HTR_PC2	0.177	0.114	1.554	0.129	0.310
	HTR_PC3	-0.164	0.076	-2.152	0.037	0.160
	HTR_PC4	0.024	0.101	0.237	0.814	0.888
	HTR_PC5	0.081	0.106	0.765	0.449	0.770
	HTR_PC6	-0.030	0.088	-0.336	0.739	0.887
	HTT_PC1	-0.127	0.113	-1.127	0.268	0.536
	HTT_PC2	-0.089	0.144	-0.619	0.540	0.810
	HTT_PC3	-0.160	0.096	-1.661	0.106	0.310
	HTT_PC4	0.044	0.096	0.460	0.648	0.864
	**HTT_PC5**	**-0.213**	**0.075**	**-2.830**	**0.007**	**0.084**
	HTT_PC6	-0.010	0.091	-0.112	0.911	0.911
Reactivity	HTR_PC1	-0.083	0.106	-0.783	0.439	0.878
	HTR_PC2	0.023	0.096	0.235	0.816	0.880
	HTR_PC3	-0.107	0.074	-1.447	0.157	0.628
	HTR_PC4	0.134	0.086	1.567	0.126	0.628
	HTR_PC5	-0.025	0.090	-0.278	0.783	0.880
	HTR_PC6	-0.161	0.063	-2.566	0.014	0.168
	HTT_PC1	-0.017	0.095	-0.181	0.858	0.880
	HTT_PC2	-0.019	0.122	-0.152	0.880	0.880
	HTT_PC3	-0.083	0.081	-1.027	0.312	0.749
	HTT_PC4	-0.102	0.081	-1.255	0.218	0.654
	HTT_PC5	-0.018	0.076	-0.243	0.809	0.880
	HTT_PC6	0.046	0.077	0.602	0.551	0.880
Agreeableness	HTR_PC1	-0.106	0.054	-1.958	0.056	0.224
	**HTR_PC2**	**0.148**	**0.050**	**2.938**	**0.005**	**0.060**
	HTR_PC3	-0.035	0.058	-0.604	0.550	0.847
	HTR_PC4	0.089	0.067	1.339	0.189	0.454
	HTR_PC5	0.010	0.070	0.138	0.891	0.972
	HTR_PC6	0.024	0.058	0.417	0.679	0.847
	HTT_PC1	-0.028	0.074	-0.380	0.706	0.847
	HTT_PC2	-0.049	0.095	-0.519	0.607	0.847
	HTT_PC3	-0.101	0.063	-1.589	0.121	0.363
	HTT_PC4	0.053	0.064	0.829	0.413	0.826
	HTT_PC5	0.001	0.059	0.023	0.982	0.982
	HTT_PC6	0.120	0.060	1.999	0.053	0.224
Extraversion	HTR_PC1	-0.067	0.107	-0.631	0.532	0.919
	HTR_PC2	0.014	0.097	0.141	0.889	0.919
	HTR_PC3	-0.104	0.074	-1.398	0.171	0.919
	HTR_PC4	0.026	0.086	0.305	0.762	0.919
	HTR_PC5	-0.009	0.090	-0.102	0.919	0.919
	HTR_PC6	-0.018	0.075	-0.235	0.815	0.919
	HTT_PC1	-0.084	0.096	-0.880	0.385	0.919
	HTT_PC2	0.110	0.123	0.899	0.375	0.919
	HTT_PC3	-0.017	0.082	-0.207	0.837	0.919
	HTT_PC4	-0.028	0.082	-0.339	0.736	0.919
	HTT_PC5	-0.014	0.076	-0.182	0.856	0.919
	HTT_PC6	0.140	0.077	1.808	0.079	0.919
Openness	HTR_PC1	-0.168	0.103	-1.637	0.111	0.666

	HTR_PC2	0.105	0.093	1.132	0.265	0.668
	HTR_PC3	-0.001	0.072	-0.017	0.986	0.986
	HTR_PC4	-0.022	0.083	-0.270	0.789	0.861
	HTR_PC5	0.085	0.087	0.978	0.335	0.668
	HTR_PC6	0.043	0.072	0.593	0.557	0.668
	HTT_PC1	-0.077	0.092	-0.839	0.407	0.668
	HTT_PC2	-0.093	0.118	-0.786	0.437	0.668
	HTT_PC3	0.056	0.078	0.719	0.477	0.668
	HTT_PC4	0.053	0.079	0.670	0.507	0.668
	HTT_PC5	-0.153	0.060	-2.562	0.014	0.168
	HTT_PC6	-0.088	0.074	-1.185	0.244	0.668

Est = estimate, SE = Standard error, t = t-value, p = p value, boldface indicates p-value approaches significance

Table S5: Model statistics for *HTR1A* and *SLC6A4* CpG individual CpG methylation scores and their association with Dominance scores in chimpanzees

	cg probe	Est	SE	t	p	p_{adj}	sign
HTR1A	cg13666507	5.054	3.856	1.311	0.201	0.603	
	cg08259925	2.248	6.509	0.345	0.733	0.855	
	cg16280141	2.809	4.313	0.651	0.521	0.855	
	cg07839533	-6.201	3.816	-1.625	0.116	0.487	
	cg16807523	0.872	3.651	0.239	0.813	0.899	
	cg11432303	4.450	4.766	0.934	0.359	0.685	
	cg17386123	10.192	4.979	2.047	0.051	0.357	
	cg04694812	5.279	4.299	1.228	0.231	0.606	
	cg10198270	-12.668	7.718	-1.641	0.113	0.487	
	cg11615755	1.758	4.248	0.414	0.682	0.855	
	cg08764163	5.965	2.007	2.972	0.005	0.053	
	cg04427003	-2.141	5.703	-0.375	0.710	0.855	
	cg02266732	5.980	6.333	0.944	0.354	0.685	
	cg27420687	1.812	3.006	0.603	0.552	0.855	
	cg04799838	-1.440	3.666	-0.393	0.698	0.855	
	cg20598238	-5.362	3.866	-1.387	0.177	0.603	
	cg15092168	0.224	6.431	0.035	0.972	0.978	
	cg10588470	-0.085	2.998	-0.028	0.978	0.978	
	**cg23448729**	**-6.611**	**1.686**	**-3.920**	**0.0003**	**0.006**	**
	cg27615388	-5.663	4.933	-1.148	0.261	0.609	
	cg09698471	-3.801	7.073	-0.537	0.596	0.855	
SLC6A4	cg16647683	-3.318	10.009	-0.331	0.744	0.970	
	cg00386645	8.135	9.090	0.895	0.381	0.856	
	cg08743901	-0.287	4.902	-0.059	0.954	0.991	
	cg10146136	-2.893	10.325	-0.280	0.782	0.970	
	cg14312898	-6.088	9.566	-0.636	0.532	0.962	
	cg20209182	-10.085	7.873	-1.281	0.215	0.856	
	cg22584138	-2.775	3.177	-0.874	0.393	0.856	
	cg26438554	-5.409	14.666	-0.369	0.716	0.970	
	cg01330016	2.680	12.326	0.217	0.830	0.970	
	cg05016953	-4.494	14.337	-0.313	0.757	0.970	
	cg20592995	-4.470	7.742	-0.577	0.570	0.962	
	cg25725890	-0.133	17.481	-0.008	0.994	0.994	
	cg09921370	3.445	2.902	1.187	0.249	0.856	
	cg18584905	1.622	9.231	0.176	0.862	0.970	
	cg10241426	11.713	13.985	0.838	0.412	0.856	
	cg01991100	7.804	8.630	0.904	0.377	0.856	
	cg27427014	3.343	5.709	0.586	0.565	0.962	
	cg05951817	-2.373	2.605	-0.911	0.373	0.856	
	cg03363743	8.313	6.142	1.353	0.191	0.856	
	cg06373684	8.314	17.168	0.484	0.633	0.970	
	cg03829016	3.489	14.104	0.247	0.807	0.970	
	cg06961290	-9.729	8.114	-1.199	0.245	0.856	
	cg12074493	-7.497	7.991	-0.938	0.359	0.856	
	cg06841846	9.248	7.082	1.306	0.206	0.856	
	cg14692377	6.218	6.809	0.913	0.372	0.856	
	**cg10901968**	**-50.871**	**14.475**	**-3.514**	**0.001**	**0.027**	*
	cg26741280	-1.643	15.875	-0.104	0.919	0.991	

* indicates p<0.05, ** indicates p<0.01 (after FDR correction for multiple testing)

Table S6: Model statistics for *HTR1A* and *SLC6A4* individual CpG methylation scores and their association with Agreeableness scores in chimpanzees

	cg probe	Est	SE	t	p	p_{adj}	sign
HTR1A	cg13666507	-0.825	2.269	-0.364	0.719	0.965	
	cg08259925	0.872	3.830	0.228	0.822	0.965	
	cg16280141	-6.088	2.119	-2.874	0.006	0.126	
	cg07839533	-0.107	2.245	-0.048	0.962	0.990	
	cg16807523	-0.027	2.148	-0.013	0.990	0.990	
	cg11432303	3.848	1.838	2.093	0.043	0.151	
	cg17386123	4.266	1.856	2.299	0.027	0.151	
	cg04694812	-4.236	1.762	-2.404	0.021	0.151	
	cg10198270	-4.292	4.541	-0.945	0.353	0.674	
	cg11615755	-2.700	2.499	-1.080	0.290	0.609	
	cg08764163	-0.413	1.998	-0.207	0.838	0.965	
	cg04427003	3.652	3.355	1.089	0.286	0.609	
	cg02266732	-2.693	3.727	-0.723	0.476	0.769	
	cg27420687	2.101	1.769	1.188	0.246	0.609	
	cg04799838	-2.464	2.157	-1.142	0.264	0.609	
	cg20598238	-0.759	2.275	-0.334	0.741	0.965	
	cg15092168	-5.734	2.541	-2.256	0.029	0.151	
	cg10588470	-0.285	1.764	-0.162	0.873	0.965	
	cg23448729	1.428	1.968	0.726	0.474	0.769	
	cg27615388	-0.765	2.902	-0.264	0.794	0.965	
	cg09698471	4.337	2.041	2.125	0.040	0.151	
SLC6A4	cg16647683	-2.589	5.003	-0.518	0.610	0.871	
	cg00386645	2.192	4.543	0.483	0.635	0.871	
	cg08743901	-1.923	2.450	-0.785	0.442	0.871	
	cg10146136	-8.705	5.161	-1.687	0.107	0.413	
	**cg14312898**	**8.298**	**1.950**	**4.255**	**0.0001**	**0.003**	**
	cg20209182	2.234	3.935	0.568	0.577	0.871	
	**cg22584138**	**2.445**	**0.798**	**3.064**	**0.004**	**0.022**	*
	cg26438554	8.326	7.331	1.136	0.270	0.729	
	cg01330016	-1.558	6.161	-0.253	0.803	0.903	
	**cg05016953**	**13.141**	**3.277**	**4.010**	**0.0003**	**0.003**	**
	cg20592995	2.744	3.870	0.709	0.487	0.871	
	cg25725890	-5.757	8.738	-0.659	0.518	0.871	
	cg09921370	-0.678	1.450	-0.468	0.645	0.871	
	cg18584905	0.609	4.614	0.132	0.896	0.924	
	cg10241426	4.632	6.990	0.663	0.515	0.871	
	cg01991100	-0.418	4.313	-0.097	0.924	0.924	
	cg27427014	1.160	2.853	0.407	0.689	0.886	
	cg05951817	0.460	1.302	0.353	0.728	0.893	
	**cg03363743**	**-4.298**	**1.484**	**-2.896**	**0.006**	**0.027**	*
	cg06373684	-9.973	8.581	-1.162	0.259	0.729	
	cg03829016	-1.189	7.050	-0.169	0.868	0.924	
	cg06961290	6.313	4.056	1.557	0.135	0.456	
	cg12074493	3.151	3.994	0.789	0.439	0.871	
	cg06841846	-0.899	3.540	-0.254	0.802	0.903	
	cg14692377	1.885	3.403	0.554	0.586	0.871	
	**cg10901968**	**-36.226**	**8.764**	**-4.133**	**0.0002**	0.003	**
	**cg26741280**	**14.496**	**4.509**	**3.215**	**0.0025**	0.017	*

Table S7: Model statistics for *HTR1A* and *SLC6A4* CpG individual cpg scores and their association with Openness scores in chimpanzees

	cg probe	Est	SE	t	p	p_{adj}	sign
HTR1A	cg13666507	-0.415	2.861	-0.145	0.886	0.993	
	cg08259925	3.640	4.829	0.754	0.458	0.962	
	cg16280141	-3.542	3.200	-1.107	0.278	0.834	
	cg07839533	4.074	2.831	1.439	0.162	0.680	
	cg16807523	0.468	2.708	0.173	0.864	0.993	
	cg11432303	-0.186	3.536	-0.053	0.958	0.993	
	cg17386123	4.698	3.694	1.272	0.215	0.753	
	cg04694812	0.199	3.190	0.062	0.951	0.993	
	cg10198270	0.164	5.726	0.029	0.977	0.993	
	cg11615755	-5.439	3.152	-1.726	0.096	0.530	
	cg08764163	2.210	2.520	0.877	0.389	0.962	
	cg04427003	1.684	4.231	0.398	0.694	0.993	
	**cg02266732**	**8.676**	**2.784**	**3.117**	**0.003**	**0.032**	*
	cg27420687	-0.022	2.230	-0.010	0.992	0.993	
	cg04799838	2.155	2.720	0.792	0.435	0.962	
	cg20598238	1.310	2.868	0.457	0.652	0.993	
	cg15092168	-8.108	4.772	-1.699	0.101	0.530	
	cg10588470	-0.021	2.224	-0.009	0.993	0.993	
	cg23448729	-1.309	2.481	-0.528	0.602	0.993	
	**cg27615388**	**-5.211**	**1.628**	**-3.201**	**0.003**	**0.032**	*
	cg09698471	0.116	5.247	0.022	0.983	0.993	
SLC6A4	cg16647683	-4.942	7.943	-0.622	0.541	0.994	
	cg00386645	10.690	7.213	1.482	0.154	0.994	
	cg08743901	1.824	3.890	0.469	0.644	0.994	
	cg10146136	-7.630	8.193	-0.931	0.363	0.994	
	cg14312898	-6.883	7.591	-0.907	0.375	0.994	
	cg20209182	-7.696	6.248	-1.232	0.232	0.994	
	cg22584138	-2.796	2.521	-1.109	0.280	0.994	
	cg26438554	1.800	11.638	0.155	0.879	0.994	
	cg01330016	5.335	9.781	0.545	0.591	0.994	
	cg05016953	-2.058	11.377	-0.181	0.858	0.994	
	cg20592995	-2.498	6.143	-0.407	0.689	0.994	
	cg25725890	-14.636	7.042	-2.079	0.043	0.581	
	cg09921370	0.832	2.303	0.361	0.722	0.994	
	cg18584905	-4.173	7.325	-0.570	0.575	0.994	
	cg10241426	3.382	11.098	0.305	0.764	0.994	
	cg01991100	6.751	6.848	0.986	0.336	0.994	
	cg27427014	-0.157	4.530	-0.035	0.973	0.994	
	cg05951817	0.307	2.067	0.149	0.883	0.994	
	cg03363743	2.356	4.874	0.483	0.634	0.994	
	cg06373684	1.342	13.623	0.099	0.923	0.994	
	cg03829016	-0.083	11.192	-0.007	0.994	0.994	
	cg06961290	1.126	6.438	0.175	0.863	0.994	
	cg12074493	3.141	6.341	0.495	0.626	0.994	
	cg06841846	-1.828	5.620	-0.325	0.748	0.994	
	cg14692377	2.897	5.403	0.536	0.598	0.994	
	cg10901968	-30.545	11.179	-2.732	0.009	0.243	
	cg26741280	-1.175	12.597	-0.093	0.927	0.994	

* indicates p<0.05 (after FDR correction for multiple testing)

Table S8: Model statistics for *HTR1A* and *SLC6A4* CpG individual cpg scores and their association with Reactivity scores in chimpanzees

	cg probe	Est	SE	t	p	p_{adj}	sign
HTR1A	cg13666507	4.058	1.566	2.591	0.013	0.063	
	cg08259925	-0.822	4.714	-0.174	0.863	0.863	
	cg16280141	6.665	2.566	2.598	0.013	0.063	
	cg07839533	-2.063	2.764	-0.746	0.462	0.693	
	cg16807523	-4.549	2.091	-2.175	0.036	0.126	
	cg11432303	-5.828	2.286	-2.549	0.015	0.063	
	cg17386123	6.489	2.504	2.592	0.013	0.063	
	cg04694812	2.642	3.114	0.849	0.404	0.653	
	cg10198270	-3.121	5.590	-0.558	0.581	0.813	
	cg11615755	4.057	3.077	1.319	0.199	0.464	
	cg08764163	3.084	1.514	2.037	0.048	0.144	
	cg04427003	0.983	4.130	0.238	0.814	0.855	
	cg02266732	4.489	4.587	0.979	0.337	0.590	
	cg27420687	-0.671	2.177	-0.308	0.760	0.840	
	cg04799838	-3.242	2.655	-1.221	0.233	0.489	
	cg20598238	-1.322	2.800	-0.472	0.641	0.818	
	cg15092168	5.312	4.658	1.140	0.265	0.506	
	cg10588470	-0.797	2.171	-0.367	0.717	0.837	
	cg23448729	1.069	2.422	0.442	0.662	0.818	
	**cg27615388**	**-7.228**	**1.640**	**-4.407**	**0.0001**	**0.002**	**
	cg09698471	-9.503	5.123	-1.855	0.075	0.197	
SLC6A4	cg16647683	-2.810	8.866	-0.317	0.755	0.932	
	cg00386645	9.269	8.051	1.151	0.263	0.822	
	cg08743901	3.815	4.342	0.878	0.390	0.932	
	cg10146136	-0.785	9.146	-0.086	0.932	0.932	
	cg14312898	-4.901	8.473	-0.578	0.570	0.932	
	cg20209182	-8.656	6.974	-1.241	0.229	0.822	
	cg22584138	-0.814	2.814	-0.289	0.775	0.932	
	cg26438554	-17.251	12.991	-1.328	0.199	0.822	
	cg01330016	-2.379	10.918	-0.218	0.830	0.932	
	cg05016953	-24.391	12.700	-1.921	0.069	0.822	
	cg20592995	-2.089	6.857	-0.305	0.764	0.932	
	cg25725890	-1.760	15.484	-0.114	0.911	0.932	
	cg09921370	2.996	2.570	1.166	0.258	0.822	
	cg18584905	5.239	8.177	0.641	0.529	0.932	
	cg10241426	4.642	12.388	0.375	0.712	0.932	
	cg01991100	1.147	7.644	0.150	0.882	0.932	
	cg27427014	0.911	5.057	0.180	0.859	0.932	
	cg05951817	-2.276	2.308	-0.986	0.336	0.907	
	cg03363743	6.119	5.441	1.125	0.274	0.822	
	cg06373684	19.159	15.207	1.260	0.222	0.822	
	cg03829016	3.702	12.493	0.296	0.770	0.932	
	cg06961290	-14.380	7.187	-2.001	0.059	0.822	
	cg12074493	-2.509	7.078	-0.355	0.727	0.932	
	cg06841846	2.250	6.273	0.359	0.724	0.932	
	cg14692377	7.821	6.031	1.297	0.210	0.822	
	cg10901968	-18.198	32.040	-0.568	0.576	0.932	
	cg26741280	-2.850	14.062	-0.203	0.841	0.932	

** indicates p<0.01 (after FDR correction for multiple testing)

Table S9: Model statistics for *HTR1A* and *SLC6A4* CpG individual cpg scores and their association with Extraversion scores in chimpanzees

	cg probe	Est	SE	t	p	p_{adj}	sign
HTR1A	cg13666507	1.829	3.245	0.564	0.578	0.934	
	cg08259925	-1.137	5.478	-0.208	0.837	0.974	
	cg16280141	-3.105	3.630	-0.855	0.400	0.933	
	cg07839533	2.438	3.212	0.759	0.455	0.934	
	cg16807523	-0.305	3.072	-0.099	0.922	0.974	
	cg11432303	0.182	4.011	0.045	0.964	0.974	
	cg17386123	1.475	4.191	0.352	0.728	0.974	
	cg04694812	2.162	3.618	0.597	0.555	0.934	
	cg10198270	-1.001	6.495	-0.154	0.879	0.974	
	cg11615755	-3.152	3.575	-0.882	0.386	0.933	
	cg08764163	-3.823	2.858	-1.338	0.193	0.933	
	cg04427003	0.285	4.799	0.059	0.953	0.974	
	cg02266732	-1.730	5.330	-0.325	0.748	0.974	
	cg27420687	1.609	2.530	0.636	0.530	0.934	
	cg04799838	-0.101	3.086	-0.033	0.974	0.974	
	cg20598238	3.764	1.461	2.576	0.013	0.273	
	cg15092168	-5.945	5.413	-1.098	0.282	0.933	
	cg10588470	2.404	2.523	0.953	0.349	0.933	
	cg23448729	-2.507	1.222	-2.051	0.046	0.483	
	cg27615388	-3.817	4.151	-0.919	0.366	0.933	
	cg09698471	6.404	5.953	1.076	0.292	0.933	
SLC6A4	cg16647683	-10.882	8.829	-1.233	0.232	0.947	
	cg00386645	-2.217	8.018	-0.276	0.785	0.947	
	cg08743901	3.969	4.324	0.918	0.370	0.947	
	cg10146136	-2.987	9.108	-0.328	0.746	0.947	
	cg14312898	2.663	8.438	0.316	0.756	0.947	
	cg20209182	-6.211	6.945	-0.894	0.382	0.947	
	cg22584138	-1.089	2.802	-0.389	0.702	0.947	
	cg26438554	13.803	12.937	1.067	0.299	0.947	
	cg01330016	5.948	10.873	0.547	0.590	0.947	
	cg05016953	9.518	12.647	0.753	0.460	0.947	
	cg20592995	2.196	6.829	0.321	0.751	0.947	
	cg25725890	-9.177	15.420	-0.595	0.558	0.947	
	cg09921370	-0.173	2.560	-0.067	0.947	0.947	
	cg18584905	9.861	8.143	1.211	0.240	0.947	
	cg10241426	2.724	12.337	0.221	0.828	0.947	
	cg01991100	6.665	7.612	0.876	0.392	0.947	
	cg27427014	-1.278	5.036	-0.254	0.802	0.947	
	cg05951817	0.206	2.298	0.090	0.930	0.947	
	cg03363743	-0.391	5.418	-0.072	0.943	0.947	
	cg06373684	3.034	15.144	0.200	0.843	0.947	
	cg03829016	-8.272	12.442	-0.665	0.514	0.947	
	cg06961290	1.214	7.157	0.170	0.867	0.947	
	cg12074493	6.438	7.049	0.913	0.372	0.947	
	cg06841846	1.303	6.248	0.209	0.837	0.947	
	cg14692377	-4.524	6.006	-0.753	0.460	0.947	
	cg10901968	-34.488	31.907	-1.081	0.293	0.947	
	cg26741280	3.108	14.003	0.222	0.827	0.947	
